# New Strategies for the Functionalization of Carbonyl Derivatives via α-Umpolung: From Enolates to Enolonium Ions

**DOI:** 10.1021/acs.accounts.3c00171

**Published:** 2023-05-25

**Authors:** Philipp Spieß, Saad Shaaban, Daniel Kaiser, Nuno Maulide

**Affiliations:** Institute of Organic Chemistry, University of Vienna, Währinger Straße 38, 1090 Vienna, Austria

## Abstract

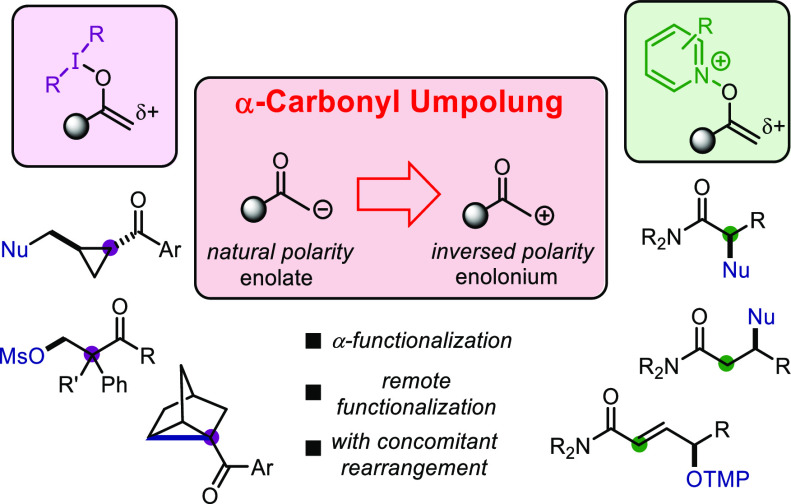

Umpolung, a term describing the reversal of innate polarity, has become an indispensable tool to unlock new chemical space by overcoming the limitations of natural polarity. Introduced by Dieter Seebach in 1979, this principle has had a tremendous impact on synthetic organic chemistry, offering previously inaccessible retrosynthetic disconnections. In contrast to the great progress made over the past decades for the generation of effective acyl anion synthons, the umpolung at the α-position of carbonyls (converting enolates into enolonium ions) has long proved challenging and only recently regained traction. Aiming to develop synthetic approaches to α-functionalization capable of complementing enolate chemistry, our group initiated, nearly 6 years ago, a program devoted to the α-umpolung of carbonyl derivatives. In this Account, following an overview of established methods, we will summarize our findings in this rapidly developing field. We focus on two distinct, yet related, topics of two carbonyl classes: (1) amides, where umpolung is enabled by electrophilic activation, and (2) ketones, where umpolung is enabled using hypervalent iodine reagents. Our group has developed several protocols to allow amide umpolung and subsequent α-functionalization, relying on electrophilic activation. Over the course of our investigations, transformations that are particularly challenging using enolate-based approaches, such as the direct α-oxygenation, α-fluorination, and α-amination of amides as well as the synthesis of 1,4-dicarbonyls from amide substrates, have been unlocked. Based on some of our most recent studies, this method has been shown to be so general that almost any nucleophile can be added to the α-position of the amide. In this Account, special emphasis will be placed on the discussion of mechanistic aspects. It is important to note that recent progress in this area has involved a shift in focus, moving even further away from the amide carbonyl, a development that shall also be detailed in a final subsection that highlights our latest investigations of umpolung-based remote functionalization of the β- and γ-positions of amides. The second section of this Account covers our more recent work dedicated to the exploration of the enolonium chemistry of ketones, unlocked through the use of hypervalent iodine reagents. By placing our work in the context of previous pioneering achievements, which mainly focused on the α-functionalization of carbonyls, we discuss new skeletal reorganizations of enolonium ions enabled by the unique properties of incipient positive charges α to electron-deficient moieties. Transformations such as intramolecular cyclopropanations and aryl migrations are covered and supplemented by detailed insight into the unusual nature of the intermediate species, including nonclassical carbocations.

## Key References

- KaiserD.; de la TorreA.; ShaabanS.; MaulideN.Metal-Free Formal Oxidative C-C Coupling by In Situ Generation of an Enolonium Species. Angew. Chem. Int. Ed.2017, 56( (21), ), 5921–592510.1002/anie.20170153828429392.^1^*First use of pyridine N-oxides for the umpolung of amides to allow intramolecular cyclization*.
- AdlerP.; TeskeyC. J.; KaiserD.; HolyM.; SitteH. H.; MaulideN.α-Fluorination of Carbonyls with Nucleophilic Fluorine. Nat. Chem.2019, 11( (4), ), 329–33410.1038/s41557-019-0215-z30833720.^2^*First directα-C–H fluorination of amides using fluoride*.
- GonçalvesC. R.; LemmererM.; TeskeyC. J.; AdlerP.; KaiserD.; MaryasinB.; GonzálezL.; MaulideN.Unified Approach to the Chemoselective α-Functionalization of Amides with Heteroatom Nucleophiles. J. Am. Chem. Soc.2019, 141( (46), ), 18437–1844310.1021/jacs.9b0695631714077PMC6879173.^3^*General method for amide umpolung with a remarkable range of nucleophiles, together with new vistas on the mechanistic pathway*.
- LiJ.; BauerA.; Di MauroG.; MaulideN.α-Arylation of Carbonyl Compounds through Oxidative C-C Bond Activation. Angew. Chem. Int. Ed.2019, 58( (29), ), 9816–981910.1002/anie.201904899PMC677153231112360.^4^*Unsual skeletal reorganization in the area of enolonium chemistry*.

## 1 Introduction

**Scheme 1:**
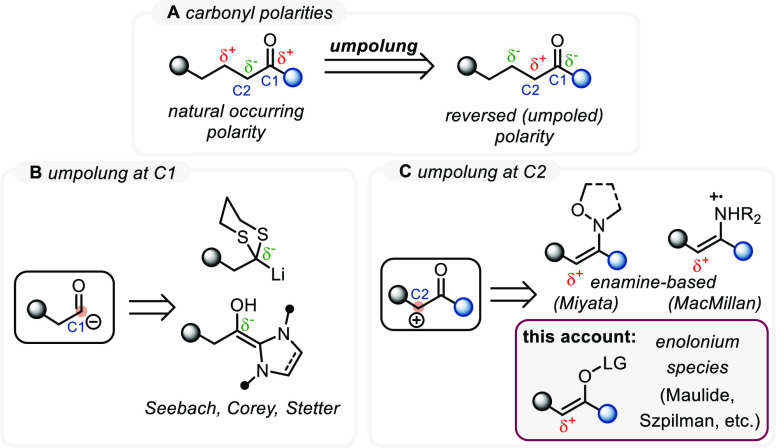
Carbonyl Polarity and State-of-the-Art Umpolung Techniques

Virtually all (retro)synthetic disconnections relying on ionic chemistry are based entirely on what is colloquially called natural polarity. This term describes the polarization of specific positions within a molecule, as determined by the electronegativity and associated inductive and mesomeric effects of neighboring functional groups. In this sense, for example, the natural polarity of a carbonyl group at C1 is δ+, indicating electrophilicity due to the electron-withdrawing properties of the adjacent oxygen (Scheme 1A). Consequently, as C1 is electrophilic, the hydrogen atoms of C2 are prone to abstraction, rendering that particular carbon nucleophilic. By virtue of vinylogy,^5^ this alternating polarity can, in theory, be extended ad infinitum, as α,β-unsaturated carbonyls are electrophilic at C3 (the β-position) and so on.

While, for example, the electrophilicity of the carbonyl C1 is one of the most fundamental reactivity principles of organic synthesis, for a long time, the inability to add carbonyl synthons to electrophiles posed a significant limitation. Although isolated reactions, such as the benzoin condensation,^6^ had already proven the possibility of polarity reversal at the carbonyl C1, it was only in the 1960s and 1970s that the advent of pioneering work by Stetter^7^ as well as Corey and Seebach,^8,9^ reporting the eponymous reactions, brought this concept to the forefront of synthetic chemistry (Scheme 1B). Umpolung, as this polarity reversal was termed by Seebach, has since played a vital role in the synthetic chemist’s toolbox and has undergone further sophisticated developments.^10−12^

In comparison to the umpolung of the C1 position of carbonyls, the umpolung at C2 is less firmly established as a useful synthetic tool (Scheme 1C). Although several approaches to C2-umpolung have been reported, such as the enamine-based strategies by Miyata and MacMillan,^13−17^ methods that simultaneously provide general solutions to this challenge and have the potential to compete with enolate chemistry (i.e., the natural polarity transformations) in terms of utility and chemoselectivity are scarce. With this in mind, we set out to apply our group’s extensive experience in the area of electrophilic amide activation to contribute to the solution of this long-standing challenge.^18,19^

## 2 Umpolung of Amides

**Scheme 2:**
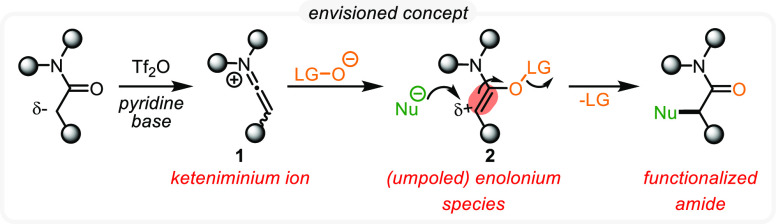
General Hypothesis for an Umpolung Concept of Amides

Well aware of the fact that highly electrophilic keteniminium species **1** can be generated chemoselectively through electrophilic amide activation, we hypothesized that their interception with *N*-oxides could trigger the formation of enolonium species **2**, postulated to exhibit significant electrophilicity at the α-position of the former carbonyl. As **2** is endowed with a leaving group on oxygen, we anticipated such structures to be susceptible to nucleophilic attack, simultaneously displacing the leaving group and restoring the amide carbonyl, thereby providing a new way of introducing nucleophiles to the α-position of amides (Scheme 2).

Notably, while similar reactivity had been previously observed independently by Hashmi, Ye, and Zhang, who generated labile N–O bonds through the addition of *N*-oxides to metal-activated alkynes,^20−22^ precedent in the context of amide activation was significantly more restricted.^23^ In fact, previous investigation was limited to a singular report by Ghosez, the pioneer of electrophilic amide activation, and co-workers. They described the formation of α,β-unsaturated amides through the treatment of α-branched amides with phosgene, followed by the addition of pyridine *N*-oxide, and notably, within this report, a sole example of α-chlorination was also shown.^24^

**Scheme 3:**
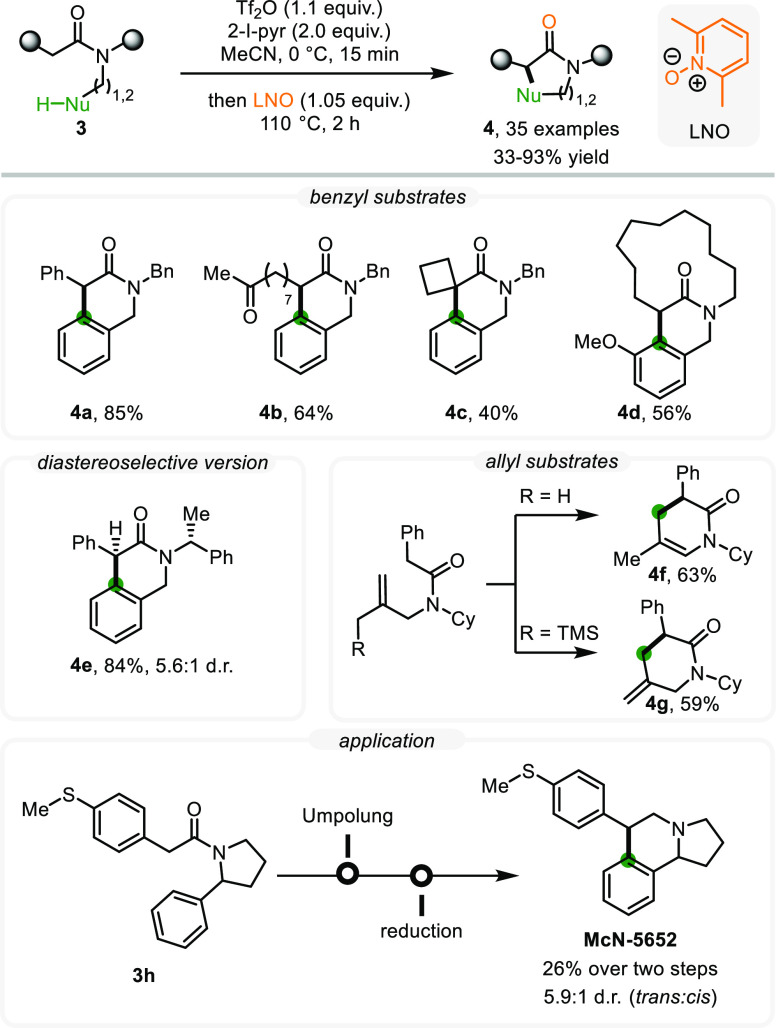
Intramolecular Oxidative C–C Coupling via Enolonium Species

At the outset, we decided to probe our hypothesis by investigating an intramolecular cyclization of *N*-benzylamides (**3**). After considerable screening, 2,6-lutidine-*N*-oxide (LNO), in combination with elevated temperature, proved to be an ideal system to enable intramolecular nucleophilic capture (Scheme 3).^1^ Functional group tolerance was broad, and several moieties, including aryl and carbonyl groups (**4a**, **4b**), were tolerated, as were amides of varying shape and constitution (exemplified by spirocyclic product **4c** and large-ring lactam **4d**).

The tolerance of a ketone carbonyl in substrate **4b** is worthy of further discussion. Indeed, electrophilic amide activation relies fundamentally on an increased electron density at the amide oxygen; therefore, in competition with other carbonyls, for example, esters or ketones, there is a very strong probability that activation takes place exclusively at the amide. This feature enables a type of chemoselectivity that is very seldom observed in carbonyl chemistry. Importantly, amides bearing a stereogenic center enabled diastereoselective cyclization (**4e**). In addition, olefins proved to be compatible nucleophiles, allowing for the formation of dihydropyridinones (**4f**) and tetrahydropyridinones (**4g**) through formal C–H-ene and C–H-Sakurai reactions. Finally, the synthetic utility of this cyclization reaction was demonstrated by the preparation of the highly potent serotonin 5-HT transporter uptake blocker (**McN-5652**) in two steps, starting from a simple amide (**3h**).

**Scheme 4:**
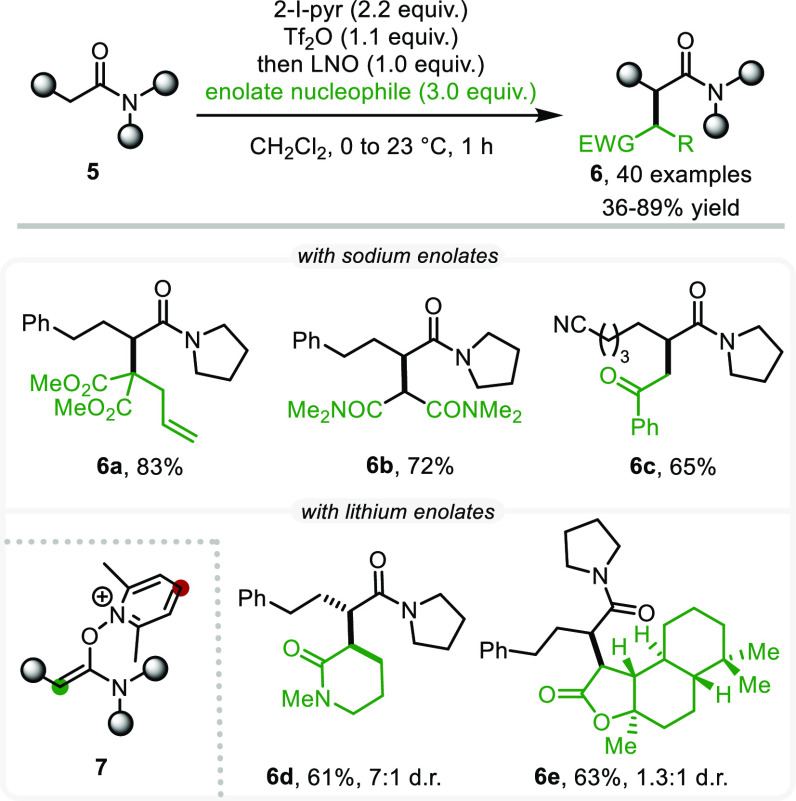
Intermolecular C–C Coupling with Enolates for the Synthesis of 1,4-Dicarbonyls

Following this initial success, we set out to unlock an intermolecular C–C bond-forming event and investigated the addition of enolates to the α-position of amides.^25^ Here, potential challenges were to be overcome in order to achieve regioselective attack on the enolonium species (green circle in intermediate **7**), with attack at the other electrophilic site (red circle in **7**)^26^ or elimination triggered by the basic reactants/reagents being the more obvious pitfalls. Pleasingly, LDA- or NaH-preformed enolates were indeed suitable nucleophiles, reacting exclusively in the desired manner, which allowed us to generalize this metal-free method for the preparation of otherwise difficult-to-access 1,4-dicarbonyl compounds (Scheme 4).^27^ The reaction tolerated a wide range of enolates, including malonates (**6a**), malonamides (**6b**), and ketones (**6c**). In addition, lactams (**6d**) and a natural-product-derived lactone (**6e**, from Sclareolide) highlighted the synthetic utility of this method.

**Scheme 5:**
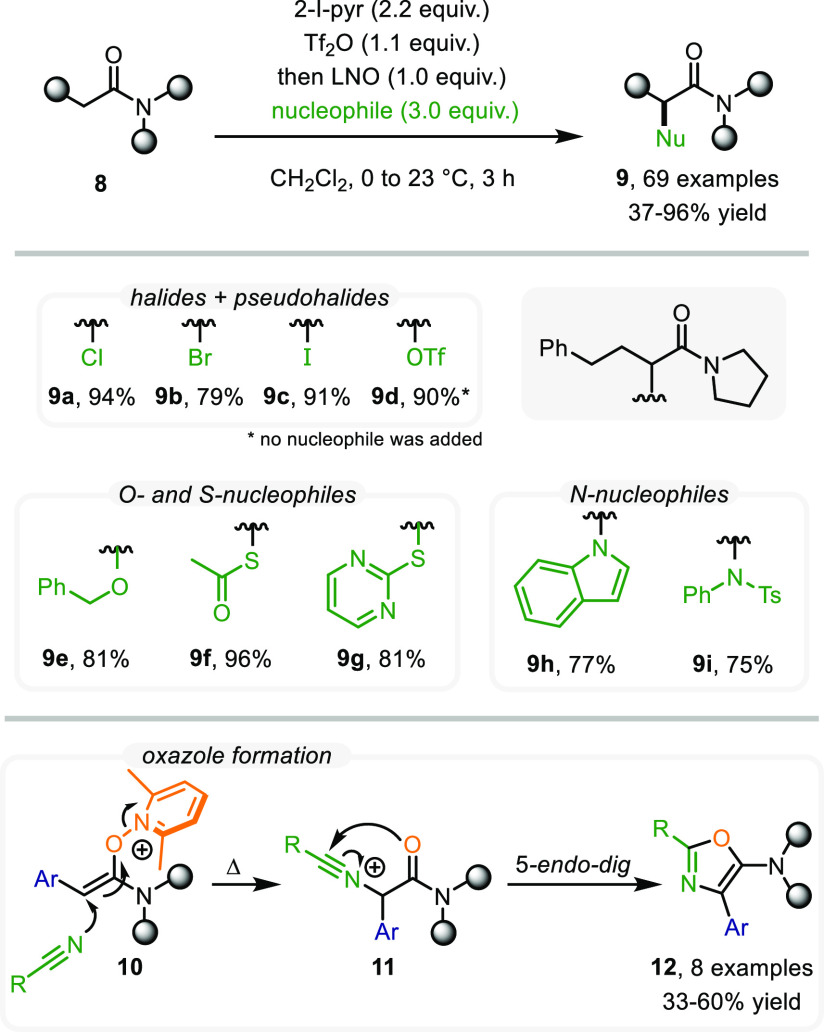
Universal Amide Umpolung Protocol

After this successful intermolecular addition, we wondered whether this concept could be further developed into a universal protocol that would allow the introduction of not only C-based but all general types of nucleophiles, including O-, N-, and S-containing compounds, as well as halogens. Without requiring adjustments to the previous reaction conditions, we were able to access α-halogenated (**9a**–**9c**), α-oxygenated (**9e**), α-thiolated (**9f**, **9g**), and α-aminated amides (**9h**, **9i**) in very good yields (Scheme 5).^3^ Unexpectedly, in the absence of an appropriate nucleophile in the umpolung event, we observed the formation of α-triflyloxy amide **9d**, which, despite its significant instability, was isolated and characterized.

In addition, this concept was used for the synthesis of heterocycles: when using nitrile solvents in the absence of other nucleophiles, solvent attack on the enolonium species took place, furnishing nitrilium ions **11** which rapidly undergo 5-endo-dig cyclization, ultimately delivering 5-aminooxazoles (**12**) in a single step (Scheme 5).^28^ Notably, however, this reactivity remained confined to α-arylamides, which we reasoned to be essential for the LUMO stabilization of enolonium species **11**.

**Scheme 6:**
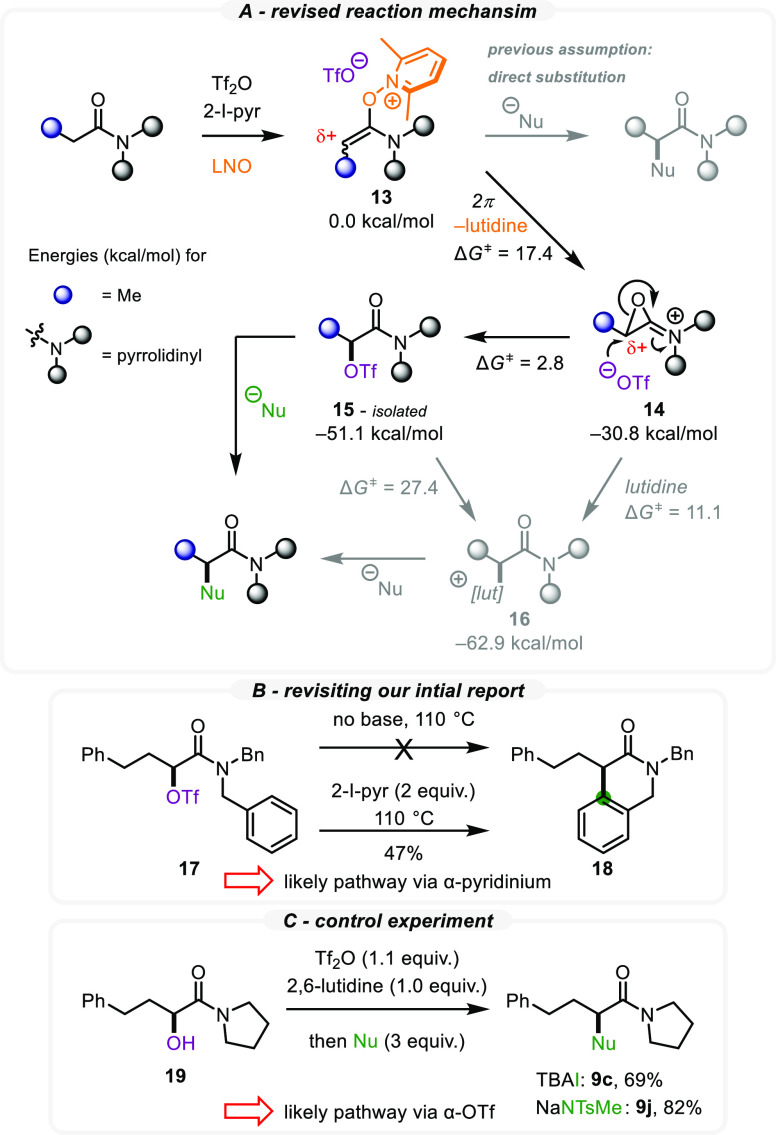
Experimental Studies and QM Calculations on the Umpolung Mechanism

The observation of α-OTf amide **9d** made us aware of possible alternative mechanistic pathways for this transformation. Therefore, quantum mechanical (QM) calculations were performed using enolonium species **13** as the starting point (Scheme 6A). Based on the obtained results, the N–O bond of the enolonium ion is most likely cleaved through a 2π-electrocyclization process with the simultaneous loss of lutidine (Δ*G* = −30.8 kcal/mol). Subsequent nucleophilic attack of the triflate on epoxide **14** (S_N_2-type attack) forms the experimentally observed α-OTf amide **15**. This process is both kinetically (Δ*G*^⧧^ = 2.8 kcal/mol) and thermodynamically favored (Δ*G* = −20.3 kcal/mol). We additionally considered the epoxide ring opening through an attack by lutidine. However, this reaction was kinetically less favorable (Δ*G*^⧧^ = 11.1 kcal/mol), as was the hypothetical S_N_2 interconversion between the triflate and lutidine adducts (Δ*G*^⧧^ = 27.4 kcal/mol). Similar processes have been calculated for the attack with 2-iodopyridine as well, which revealed a slightly lower S_N_2 interconversion barrier (4.9 kcal/mol lower than for lutidine; not shown).

Driven by these computational insights, we experimentally reinvestigated our prior umpolung-mediated intramolecular cyclization of *N*-benzyl amides, a process that benefited from higher reaction temperatures in order to obtain high yields (Scheme 6B). To probe whether this reaction also proceeds via an α-OTf amide intermediate, the α-OTf amide **17** was prepared independently. Interestingly, subjecting **17** to an increased temperature in the absence of a pyridine base did not provide the expected dihydroisoquinolin-3-one product **18**. In contrast, in the presence of 2-iodo-pyridine, the desired cyclized product was indeed obtained. It therefore transpired that the presence of a pyridinium intermediate might be crucial for this intramolecular process and the formation of the corresponding final product (**18**). Interestingly, the α-OTf amide (generated in situ from α-hydroxy amide **19**) was rapidly substituted by an iodide or nitrogen nucleophile in the absence of additional base (the lutidine being used solely as a base to enable triflation) to provide α-halogenated and α-aminated products **9c** and **9j** (Scheme 6C), thus painting a picture whereby the precise reaction pathway might be case-specific: higher temperatures and a somewhat poorer π-nucleophile (such as a tethered arene or alkene) might render the occurrence of pyridinium species more likely.

Due to the high importance of fluorinated compounds in medicinal chemistry and the challenges accompanying the introduction of that element into complex organic substrates, we set out to investigate the application of this concept to the α-fluorination of amides with fluoride (F^–^). Notably, previous elegant methods for the synthesis of α-fluorinated amides exclusively relied on electrophilic fluorinating reagents (F^+^), and none proceeded directly from unfunctionalized amide starting materials.^29−31^

**Scheme 7:**
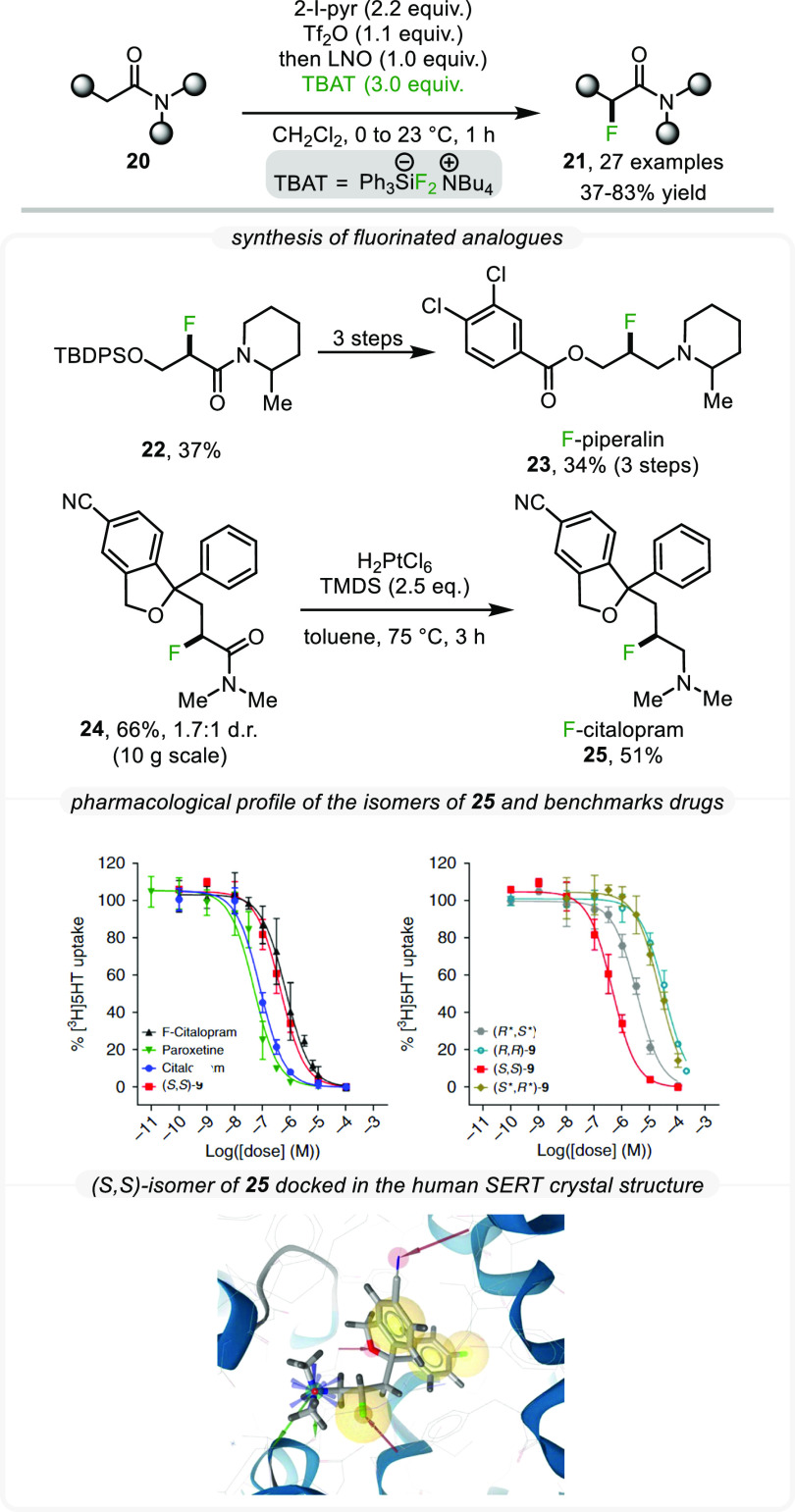
α-Fluorination of Amides and Its Application to Marketed Drugs

In our search for an optimal fluoride source harnessing our umpolung protocol, we found several reagents to give satisfactory results, with TBAT (tetrabutylammonium difluorotriphenylsilicate) providing the highest and most reproducible yields (Scheme 7).^2^ Once again, excellent functional group tolerance was observed, and a scope of 27 α-fluorinated amides was prepared. As previous studies have shown that β-fluorination of amines can improve bioavailability due to enhanced membrane permeation properties,^32,33^ we saw considerable potential in accessing these types of compounds through a reduction of the corresponding α-F-amide. Thus, fluorinated analogs of piperaline **23** (a fungicide) and citalopram **25** (an antidepressant) were successfully prepared in two to four steps from commercially available starting materials (Scheme 7).

Remarkably, the preparation of **25** using **24** was easily performed on a 10 g scale. The resulting compound, F-citalopram **25**, was evaluated in biological assays using HEK293 cells expressing human SERT. All four stereoisomers were shown to block [3H]5-HT uptake, with the (*S*,*S*)-isomer being the most active (bottom right). While the biological activity of F-citalopram was lower than that of citalopram itself (bottom left), the (S,S)-configured species was found to be the most potent of the four stereoisomers, an effect ascribed to an *in silico* predicted stereospecific interaction inside the binding pocket (inset of Scheme 7).

**Scheme 8:**
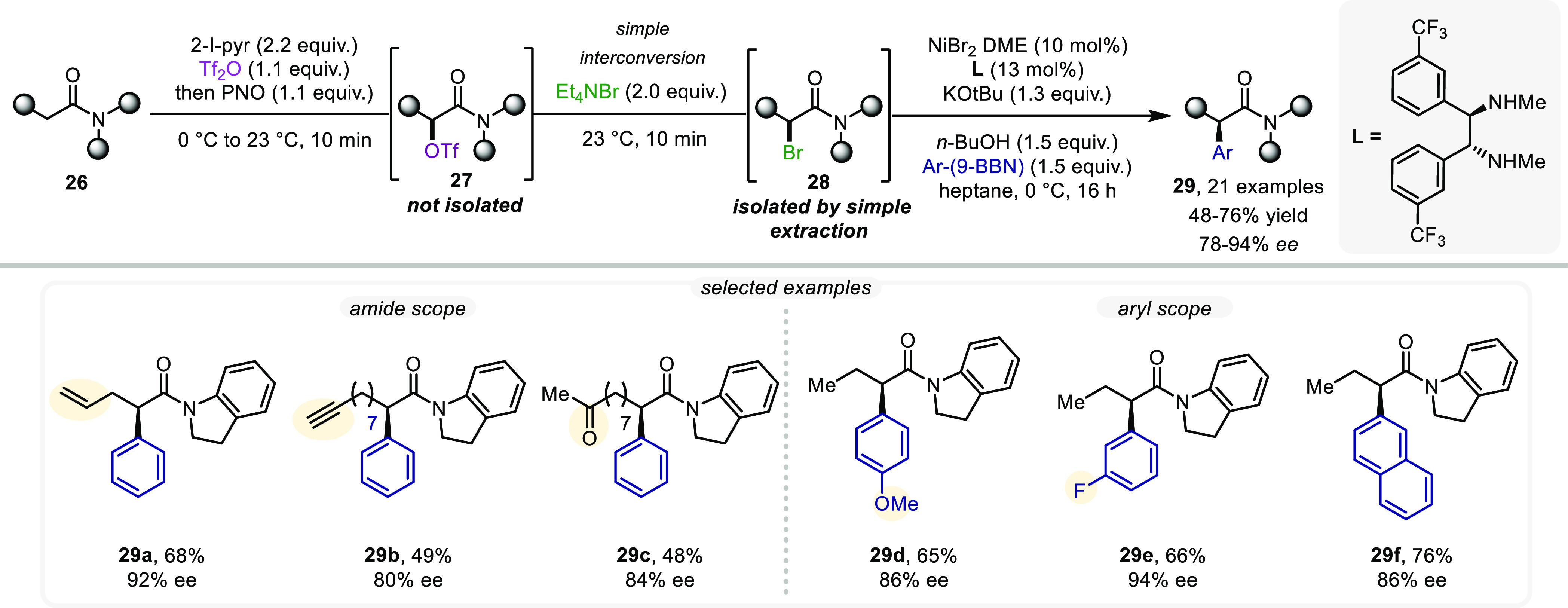
Stereoselective α-Arylation with Arylboron Reagents Enabled by Amide Umpolung

We subsequently became intrigued by the possibility of synergistically deploying amide activation (for halogenation) as a tool for catalytic asymmetric α-amide functionalization.^36^ Initially, we set out to study α-OTf-amides (**27**, Scheme 8). However, their labile nature suggested that we adapt our strategy to include intermittent conversion to the corresponding α-Br amides (**28**).^34^ This functional group interconversion could be readily accomplished within minutes using NEt_4_Br. The α-Br amides were then more compliant with catalytic processes: inspired by the work of Fu et al.,^37^ the Ni-catalyzed asymmetric C–C cross-coupling reaction with arylboron reagents, when carried out on amide substrates bearing the indolylamide backbone, delivers α-arylated products in high yields and with good *ee* values. As seen from the selected examples (Scheme 8), a range of functional groups including olefins (**29a**) and alkynes (**29b**) allowed for a successful stereoselective C–C coupling with various boranes (**29d**–**29f**) in good yields and enantioselectivities. Strikingly, carbonyls (as exemplified by **29c**), which would have been incompatible if base-promoted halogenation was used to generate the α-Br amide intermediate, were tolerated and afforded the α-arylated product in high yield and enantioselectivity.

### 2.1 Radical-Based Amide Umpolung

**Scheme 9:**
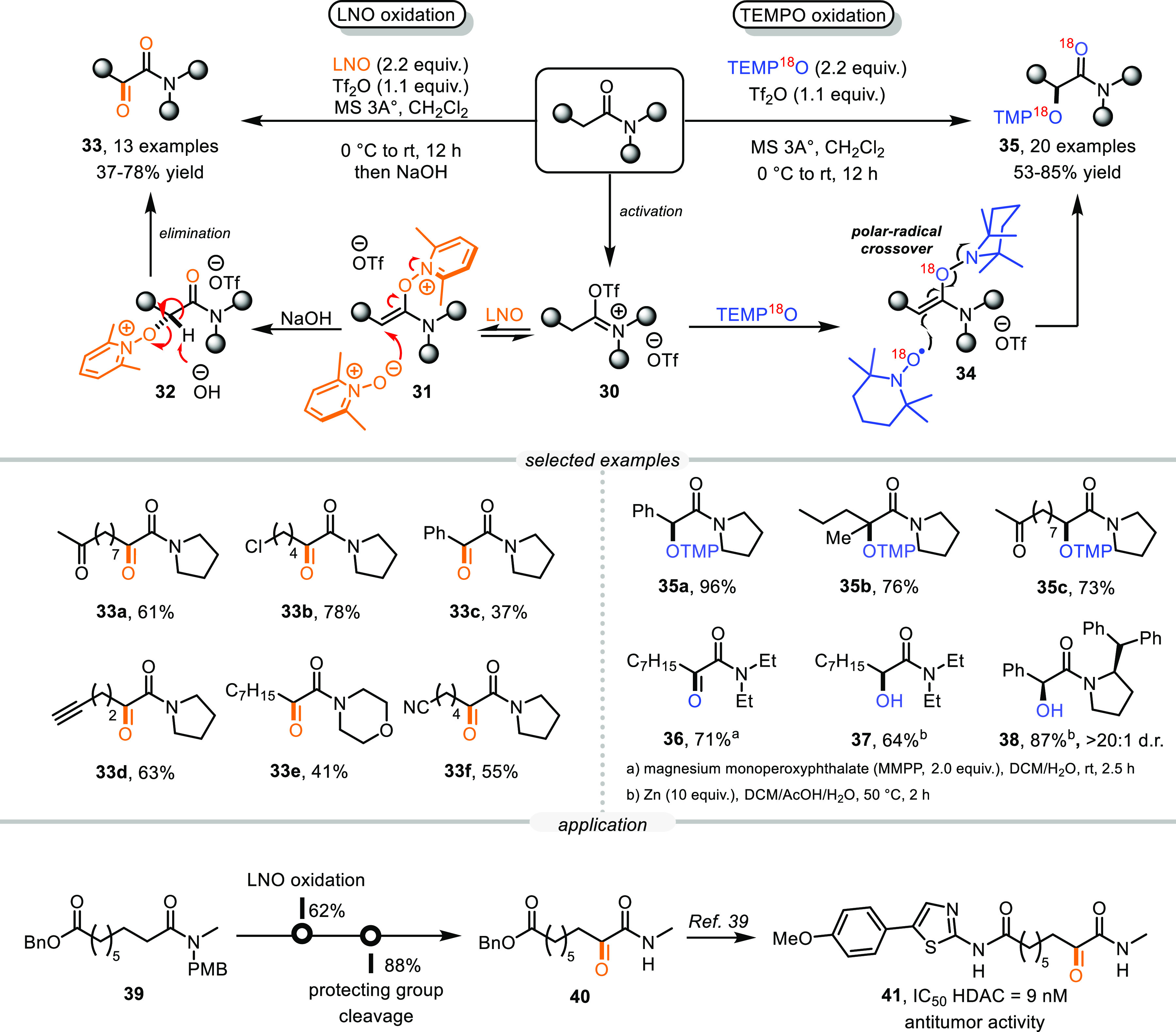
α-Oxidation of Amides to α-Keto Amides or α-Hydroxy Amides

While studying the reactivity of the enolonium species (**31**, Scheme 9), we speculated that a second equivalent of LNO could also act as a nucleophile and generate an oxapyridinium intermediate (**32**). During basic workup, **32**, possessing an acidic α-proton, could undergo an elimination step to yield α-keto amides.^35^ Indeed, increasing the LNO loading (from 1.1 to 2.2 equiv) enabled this reaction, and various α-keto amides became chemoselectively accessible in moderate yields (**33a**–**33f**). However, α-arylamides (**33c**) were found to react sluggishly under these reaction conditions. Pleasingly, however, we found that the use of TEMPO (as a substitute for LNO) allowed for an alternative path to achieve direct α-oxidation of amides, as it delivered the corresponding O-TMP adducts (**35a**–**35c**) as sole products in good yields, even for α-arylamides (**35a**). Experimental studies revealed that neither the use of ^18^O-labeled amide nor quenching with H_2_^18^O led to the incorporation of ^18^O. In contrast, ^18^O-labeled TEMPO provided the ^18^O-labeled product, with ^18^O present in both the carbonyl and the α-position. Following these studies and subsequent in-depth density functional theory (DFT) analysis,^38^ we proposed the reaction to proceed via the formation of a TEMPO-based enolonium species **34**, which in turn undergoes a polar-radical crossover reaction. In addition, reductive cleavage of the O-TMP bond could be performed to provide a free α-OH product (**37**), while the use of an enantiopure chiral amide enabled a highly diastereoselective oxidation reaction (**38**, d.r. > 20:1). Ultimately, the α-LNO oxidation protocol was successfully applied to the synthesis of a precursor compound (**40**) of a potent histone deacetylase inhibitor (**41**), tremendously shortening the previous synthetic pathway and illustrating its potential application in practice.^39^

### 2.2 Remote Functionalization Reactions Enabled by Amide Umpolung

**Scheme 10:**
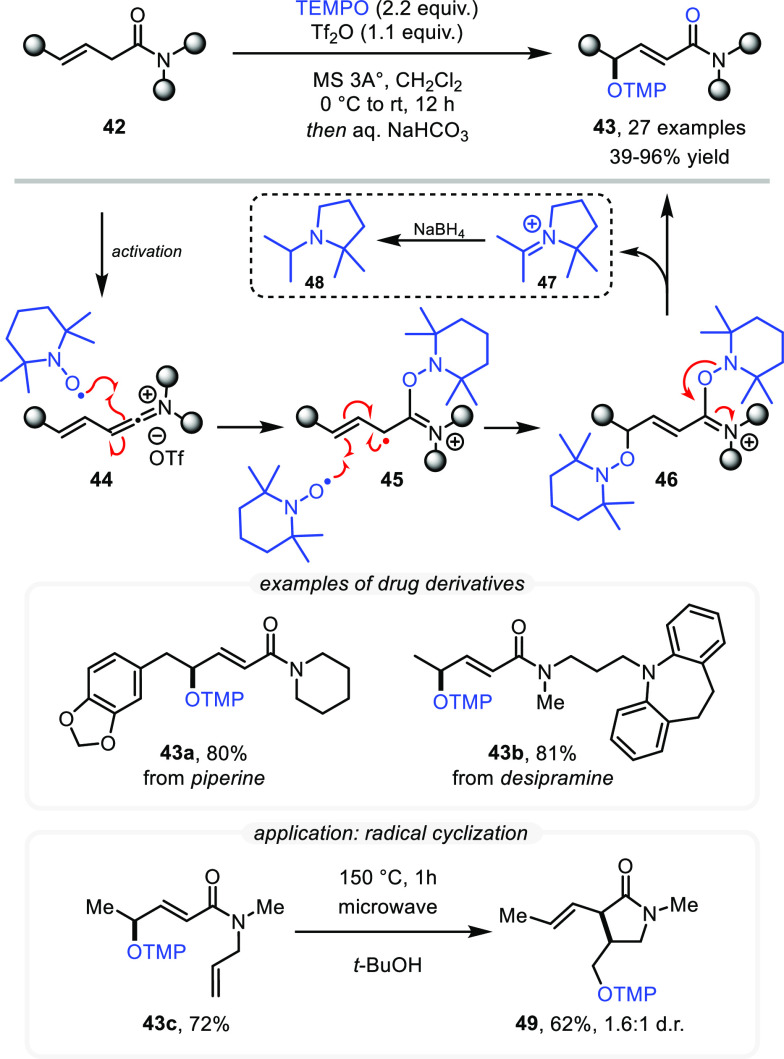
Remote γ-Oxidation of β,γ-Unsaturated Amides with TEMPO

Shortly after our communication, Kang et al. applied our method (α-OTMP amides) in the synthesis of α,β-unsaturated amides via a radical cleavage approach,^40^ clearly highlighting the practicality of our protocol and the hitherto unexplored chemical possibilities. Hoping to further extend the reach of this novel TEMPO-mediated reactivity, we applied it to a more challenging class of substrates, namely, β,γ-unsaturated amides (**39**). In contrast to the variety of methods for the α-functionalization of amides, transformations enabling access to more remote positions have been less widely explored. Exploiting our mechanistic understanding of TEMPO-based amide functionalization and applying a slightly modified protocol to β,γ-unsaturated amides, we were able to achieve the chemoselective γ-oxidation of such substrates, including complex drug derivatives (**43a** and **43b**) (Scheme 10).^41^ The proposed mechanism involves the highly stabilized allylic radical intermediate **45**, which is trapped by a second equivalent of TEMPO to give **46**.

Based on indirect experimental evidence for the formation of iminium ion **47** (including isolation of the corresponding amine **48** after reduction) in conjunction with our previous computational study,^38^ a subsequent fragmentation of **46** is thought to lead to the final reaction products.

**Scheme 11:**
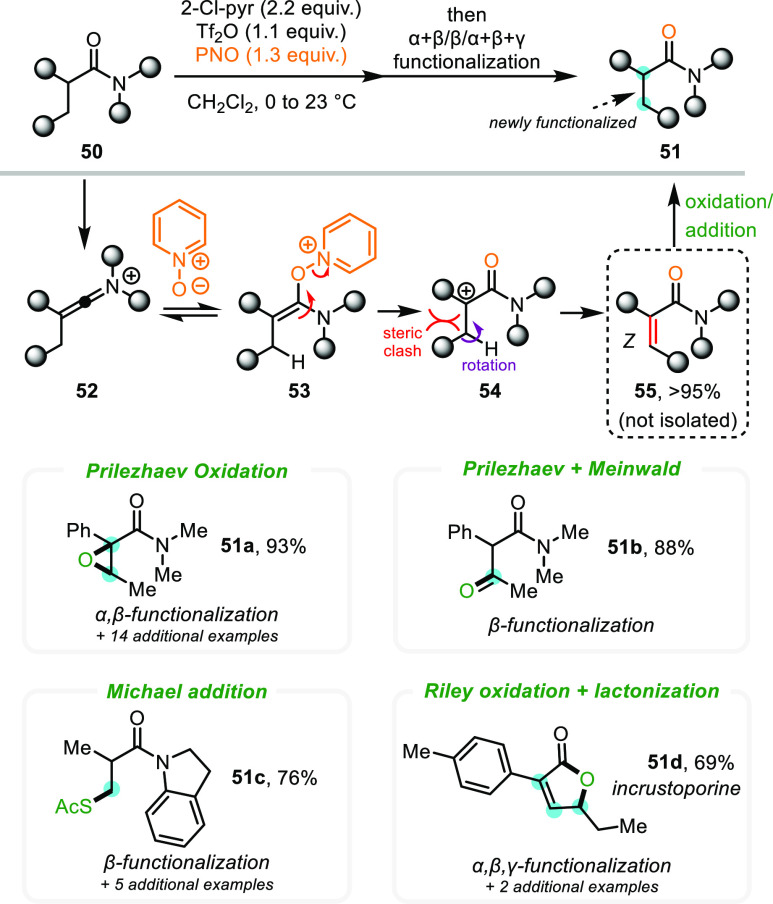
Remote Oxidations via α,β-Unsaturated Amide Intermediates

To further demonstrate the synthetic utility of the products of this reaction, we exploited the delocalized nature of allylic radicals, which can be revealed from the prepared allylic O-TMP compounds through thermally induced homolysis. For example, by employing *N*-allyl amides, we were able to induce radical cyclization reactions of substrates such as **43c** to afford γ-lactam **49** in good yield and a moderate diastereomeric ratio. Complementing the radical remote functionalization approach detailed above, we also found polar pathways to enable the installation of new functional groups at the β- and γ-positions. Specifically, we investigated the functionalization of α,β-unsaturated amides, accessible *in situ* from Tf_2_O-activated α-branched amides after treatment with a mixture of 2 equiv of base and 1 equiv of pyridine-*N*-oxide (PNO).^42^ The desired unsaturated amides (**55**) were easily prepared in high yields (>95%) under these reaction conditions and were formed exclusively as Z isomers (Scheme 11).

We attributed this selectivity to the restricted rotation of the carbocationic intermediate **54** prior to elimination. The intermediary α,β-unsaturated amides were directly subjected to subsequent oxidations or additions (one pot). For example, Prilezhaev oxidation provided epoxides such as **51a** in high yields (amounting to a one-pot α,β-functionalization). Further combination of this sequence with the Meinwald reaction allowed the preparation of β-ketoamides **51b**. This sequence is a useful complement to our LNO-based approach to form α-ketoamides (Scheme 9), forming a toolbox for accessing dicarbonyls at varying distances. In addition, a one-pot Michael addition allowed the preparation of β-thiolated amides (**51c**). Finally, we considered whether formal trifunctionalization was possible: subjection of the α,β-unsaturated amide to Riley oxidation, followed by lactonization, allowed the preparation of the natural product incrustoporine (**51d**) in 69% overall yield (formally an α,β,γ-trifunctionalization).

## 3 Umpolung of Ketones

**Scheme 12:**
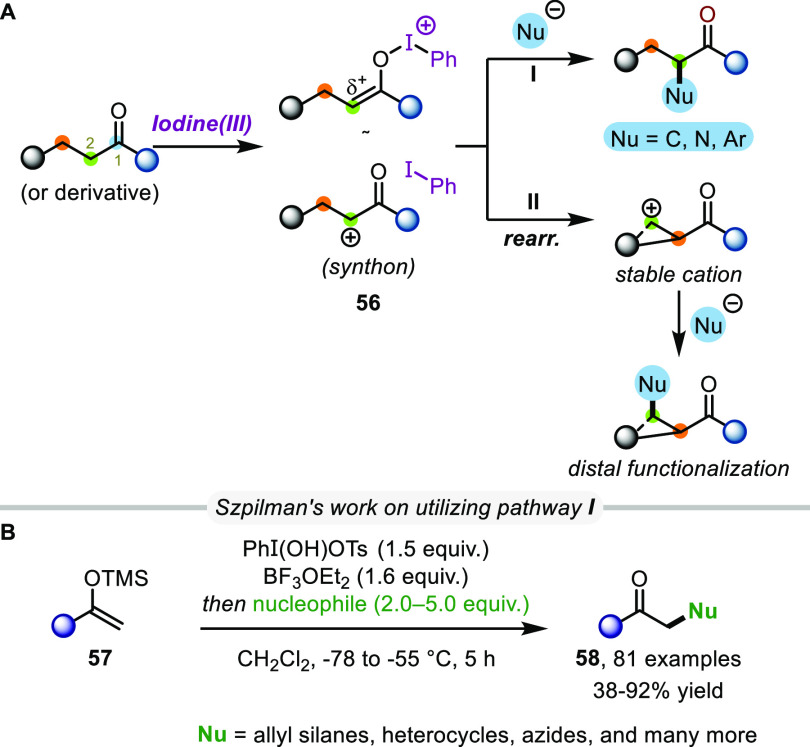
General Concept of the C2 Umpolung of Ketones with Hypervalent Iodine

Since the pioneering work of Seebach and Corey, the eponymous umpolung reactivity at the C1 of aldehydes has been extensively studied and has found its way into textbooks and introductory lectures alike. In this context, thioacetals, cyanide anions, and thiazolium-based *N*-heterocyclic carbenes (NHCs) have all proven their synthetic value. In contrast, umpolung at the C2 position of carbonyls is far less explored. Recently, the successful use of I(III) reagents to trigger the formation of enolonium species has been reported in several instances.^43^ Upon treatment of ketones (or their silyl enol ether derivatives) with hypervalent iodine reagents, the formation of α-carbocationic synthons (**56**) was observed (Scheme 12A). Notably, the addition of nucleophiles to such species enables direct α-functionalization (pathway **I**), as has been shown in many cases by Szpilman et al.^44^ In their works, the authors have employed a wide variety of nucleophiles, ranging from allyl silanes to electron-rich heterocycles^45^ and even heteroatom species, such as azides and tetrazoles (Scheme 12B).^46^ Recently, this concept was extended and successfully applied to the direct α-oxidation of ketones (umpolung of a Morita–Baylis–Hillman-type intermediate) to give α-diketones or α-tosyloxy enones (not shown).^47^

While, as described, a vast body of work exploiting pathway I had been reported, rearrangements of intermediates such as **56** prior to trapping piqued our interest, as they lead to entirely different functionalization patterns (Scheme 12, pathway **II**). The recent advances in this pathway will be discussed in more in detail in the next section.

### 3.1 α-Functionalization of Ketones with I(III) Reagents via Skeletal Rearrangement

**Scheme 13:**
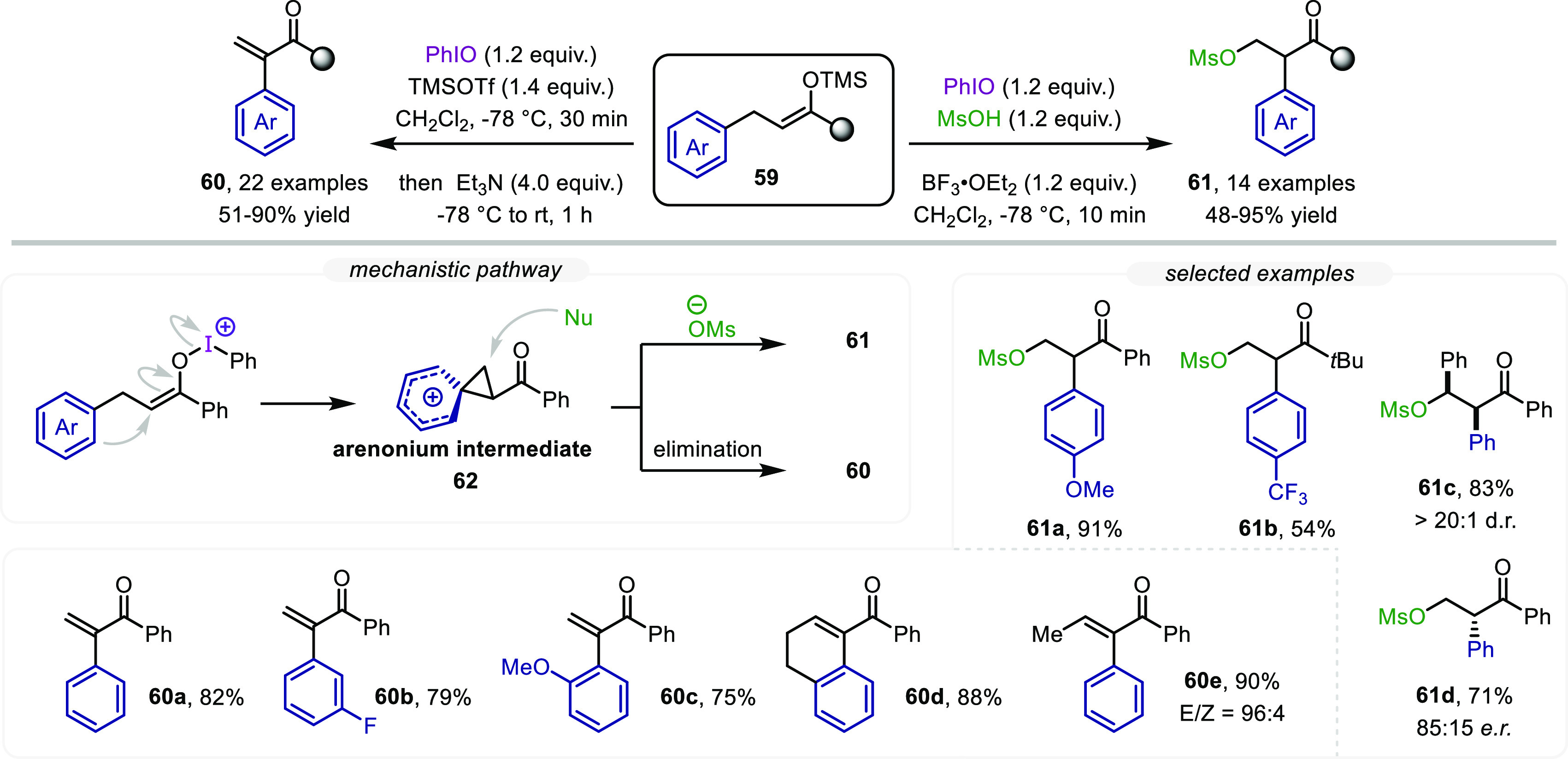
α-Arylation of Carbonyl Derivatives through 1,2-Aryl Migration/Oxidative C–C Bond Formation

**Scheme 14:**
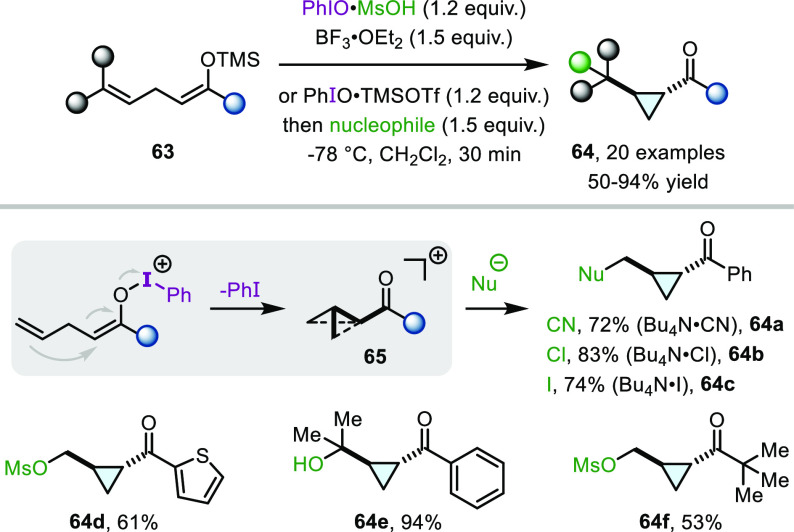
Oxidative α-Cyclopropanation of Linear Ketones

**Scheme 15:**
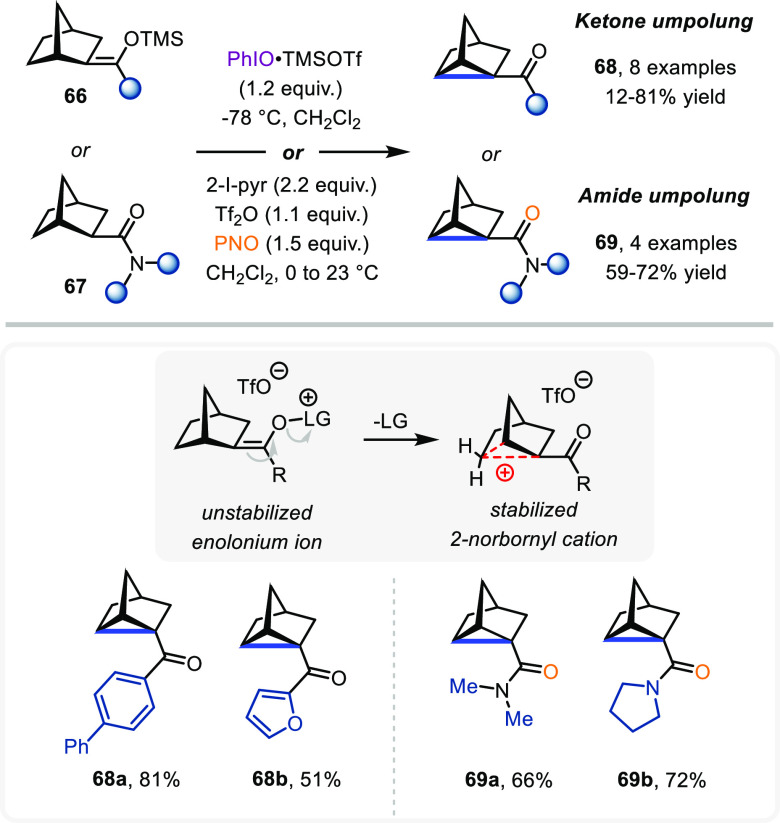
Oxidative α-Cyclopropanation of 2-Norbornyl Cations via Ketone or Amide Umpolung Strategies

In contrast to the direct trapping of enolonium species with an external nucleophile, skeletal rearrangements and migrations can also be induced (intermediate **56** in Scheme 12), particularly when carbocationic intermediates of increased stability are formed, as is the case when the allylic position of the silyl enol ether precursors is adorned with an aryl moiety (**59**, Scheme 13). Thus, after activation with a suitable iodine reagent, 1,2-aryl migration takes place via an arenonium intermediate **62**.^4^ When using methanesulfonic acid (MsOH) to generate the active I(III) species, its conjugate base is able to capture intermediate **62**, yielding α-aryl, β-methanesulfoxy ketones **61** (Scheme 13, bottom right). The transformation displays broad functional group tolerance, and the aryl group can carry either electron-donating (**61a**) or electron-withdrawing substituents (**61b**). Notably, a β,β-diphenyl-substituted ketone could be converted to product **61c** in high yield and as a single diastereomer. An asymmetric variant was also demonstrated (**61d**) when an enantioenriched hypervalent iodine reagent was employed. In contrast, employing iodosobenzene in combination with TMSOTf followed by treatment with a base resulted in the elimination of the putative *in situ*-formed β-OTf intermediate. This method provides metal-free, direct access to α-aryl enone derivatives **60** (Scheme 13, bottom).^48^ It is important to mention that β-substituted silyl enol ethers afforded adducts such as **60e** in high yield with excellent *E*/*Z* stereoselectivity. Advancing to even more challenging transformations, we have reported a protocol for cyclopropanation exploiting the propensity of certain enolonium species to engage in carbocationic rearrangements (Scheme 14).^49^ The first of these rearrangements involves the treatment of silyl enol ethers (**63**) with activated iodosobenzene at low temperatures to yield α-cyclopropyl ketones **64**. Despite the many possible competing reaction pathways, alkene-tethered substrates **63** were found to undergo rapid cyclopropanation upon oxidation, forming nonclassical carbocations (**65**) as the intermediates. Final capture by a variety of nucleophiles, such as OMs (**64d**, **64f**), halides (**64b**, **64c**), and cyanide (**64a**), was demonstrated. Interestingly, a trisubstituted alkene did not yield the expected mesylate product, but the corresponding alcohol (**64e**) was instead formed in excellent yield. The realization that the instability of putative α-carbonyl cations could be deployed to accomplish strain-inducing rearrangements encouraged us to investigate the reactivity of norbornyl ketones in this context (Scheme 15). Specifically, we believed that the incipient positive charge could favor rearrangement into the well-known and stable nonclassical 2-norbornyl cation.

Gratifyingly, we found this process to be highly efficient: an array of norbornyl-derived silyl enol ethers (**66**) furnished nortricyclene adducts (**68**) in good yields. At this stage, and bringing the concepts laid out in this Account full circle, we investigated the use of 2-norbornyl amides **67** in conjunction with our amide umpolung strategy. As shown, efficient cyclopropane formation resulted, thus highlighting the mechanistic similarities in these otherwise chemically quite different approaches.

## 4 Conclusions

Following our group’s extensive experience with the generation and functionalization of highly reactive keteniminium ions, the last 6 years have seen our efforts become increasingly focused on transposing the electrophilic center to the position α to its inception. Thus, the generation of amide-derived enolonium ions has enabled a wide range of transformations revolving around the α-functionalization of amides. As we gained a clearer perspective of the nature and reactivity of such amide-derived electrophiles, we recognized distinct similarities to enolonium ions generated through the reaction of hypervalent iodine species with silyl enol ethers.

Notably, in broadening the scope of this type of reactivity, we realized its potential to enable additional transformations, particularly in the context of rearrangements, to generate more elaborate products. At the same time, we have been able to show that the functionalization of enolonium ions and their derivatives is not necessarily limited to the α-position. Efforts toward transposing the chemistry of umpoled carbonyls to more complex settings are underway in our laboratories. These efforts also aim to address the remaining limitations sometimes observed for reactions involving the umpolung of carbons α to carbonyls, the results of the highly destabilized nature of the formed electrophilic centers, such as competition between different nucleophiles present in the reaction mixture, and reduced performance in intermolecular scenarios.

We hope that the work presented herein serves as an inspiration for other researchers to investigate the chemistry of destabilized cations. Furthermore, we anticipate that this Account will animate the synthetic community to consider umpolung reactivity as a viable alternative to more established reactions as it not only opens a path toward unprecedented scaffolds but also enables new retrosynthetic disconnections.
